# Sjogren’s Syndrome: A Series of Five Cases

**DOI:** 10.7759/cureus.71572

**Published:** 2024-10-15

**Authors:** Pravin Naphade, Shalesh Rohatgi, Prajwal Rao, Satish Nirhale

**Affiliations:** 1 Neurology, Dr. D. Y. Patil Medical College, Hospital and Research Centre, Pune, IND

**Keywords:** dystonia, miller fisher syndrome, myelin oligodendrocyte glycoprotein, neuromyelitis optica, recurrent guillain-barré syndrome, sjogren’s syndrome

## Abstract

Sjogren's syndrome is an autoimmune disorder that has a prominent involvement of exocrine glands. Systemic involvement of other organs can also happen. Peripheral nervous system involvement is common and may present as axonal sensory/sensorimotor or demyelinating polyneuropathy, mononeuritis multiplex, ganglionopathy, or cranial neuritis. It can also present with transverse myelitis, especially longitudinally extensive transverse myelitis in association with aquaporin-4 antibodies. It can also mimic central nervous system (CNS) demyelinating disorders such as multiple sclerosis. The present case series highlights some uncommon neurological presentations of Sjogren's syndrome. In this series, patients presented with Miller-Fischer syndrome, recurrent Guillain-Barre syndrome, multifocal dystonia, and demyelinating disorder of CNS. None of the cases had presenting complaints of dry mouth or eyes. Special investigations such as magnetic resonance imaging (MRI), nerve conduction studies, anti-ganglioside panel, and cerebrospinal fluid analysis, depending on the clinical presentation of the cases, were conducted. Schirmer's test, tear breakup time, antinuclear antibodies (ANA) by immunofluorescent assay, ANA blot demonstrating the presence of anti-SSA (Ro) and/or anti-SSB (La) antibodies, and lip biopsy were conducted in all cases, which confirmed the diagnosis of Sjogren's syndrome. After the diagnosis was confirmed, other tests such as C- reactive protein, serum cryoglobulin, and rheumatoid factor were conducted. Treatment was initiated with steroids, followed by long-term immunosuppression with injection rituximab. Sjogren’s syndrome may present with various presentations of neurological involvement, sometimes rare, and hence a high degree of suspicion is required when other usual causes are excluded.

## Introduction

Primary Sjogren's syndrome (SS) is a chronic autoimmune inflammatory disorder. Clinical features can be divided into two broad categories: exocrine glandular features and extraglandular features. Exocrine glandular features are characterized by diminished lacrimal and salivary gland function with resultant dryness of the eyes and mouth, whereas extraglandular features may include skin, musculoskeletal, respiratory, cardiovascular, and neurological manifestations. Neurological manifestations may include involvement of both central and peripheral nervous system. The peripheral nervous system may present with axonal sensory or sensorimotor neuropathy, small fiber neuropathy, ganglionopathy, mononeuritis multiplex, and multiple cranial neuropathies including trigeminal neuropathy or demyelinating radiculoneuropathy. Central nervous system (CNS) involvement may present as optic neuritis, asymptomatic MRI lesions, multiple sclerosis, or transverse myelitis especially longitudinally extensive transverse myelitis (LETM). Thus, clinical presentation is varied. The ACR/EULAR classification criteria for primary SS are based on a scoring system [1], and a score of 4 or more is diagnostic. Secondary SS is associated with other autoimmune diseases.

The patients can have anti-Ro/SSA antibodies with or without anti-La/SSB antibodies, a positive labial salivary gland biopsy (i.e., focal lymphocytic sialadenitis), or a systemic rheumatic disease. Anti-Ro (SSA) antibodies are not specific to SS and can be found in other autoimmune disorders including primary biliary cirrhosis; however, their presence is one of the diagnostic criteria for SS [2]. Only the presence of anti-LA (SSB) antibodies does not favor the diagnosis of SS [3]. Alternatively, the patients may have anticentromere antibodies (in the absence of systemic sclerosis) or the combination of an antinuclear antibody (ANA) ≥1:320 with a positive rheumatoid factor [4].

## Case presentation

Case 1

**Figure 1:**
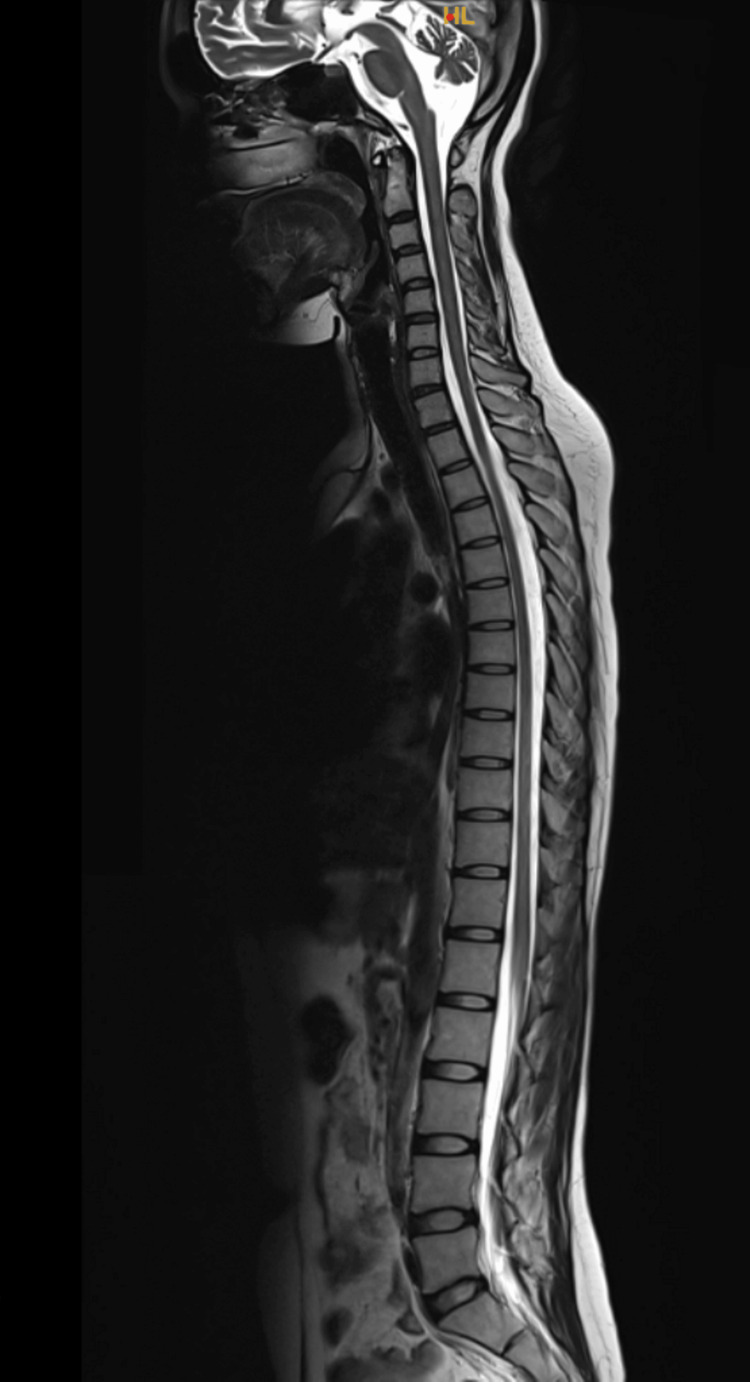
MRI of the spine, which was normal

A 40-year-old female presented with acute onset double vision, walking imbalance, and difficulty in swallowing for two days, followed by drooping of eyelids on the third day. There was bilateral ptosis, complete external ophthalmoplegia, bilateral lower motor neuron facial palsy, and weak gag reflex. Power was grade 5/5 in all four limbs, and deep tendon jerks were absent. She had sensory ataxia, and Rhomberg’s test was positive. Nerve conduction studies (Tables 1-3), cerebrospinal fluid (CSF) study, and MRI of the brain and spinal cord were unremarkable (Figure 1).

**Table 1:** Normal motor nerve conduction study

Nerve	Site	Latency (ms)	Amplitude (mV)	Velocity (m/s)
Right median	Wrist	3.2	9.1	-
	Elbow	7	9.1	55.3
Left median	Wrist	3.2	12.2	-
	Elbow	6.9	12.1	56
Right ulnar	Wrist	2.1	7.8	-
	Elbow	5.4	7.6	68.7
Left ulnar	Wrist	2.2	10.8	-
	Elbow	5.7	9.9	64.8
Right peroneal	Ankle	3.5	9.2	-
	Fibula head	8.5	8.8	58
Left peroneal	Ankle	3.9	8.2	-
	Fibula head	9.4	5.4	52.7
Right tibial	Ankle	3.6	17.9	-
	Popliteal	10	12.1	52.7
Left tibial	Ankle	2.9	9.7	-
	Popliteal	9.6	7.7	51.1

**Table 2:** Normal F waves latency

Nerve	Site	Latency (ms)
Right median	Wrist	24.3
Left median	Wrist	25.7
Right ulnar	Wrist	24.8
Left ulnar	Wrist	25
Right peroneal	Ankle	35.4
Left peroneal	Ankle	37.6
Right tibial	Ankle	40.9
Left tibial	Ankle	42.1

**Table 3:** Normal sensory nerve conduction study

Nerve	Site	Latency (ms)	Amplitude (mv)	Velocity (m/s)
Right median	Wrist	2.3	35	57
Left median	Wrist	2.4	27.2	55.1
Right ulnar	Wrist	1.7	32.8	77.4
Left ulnar	Wrist	1.6	31.9	91.5
Right sural	Leg	2.1	26.5	52.9
Left sural	Leg	1.8	25.5	77.4

**Figure 2:**
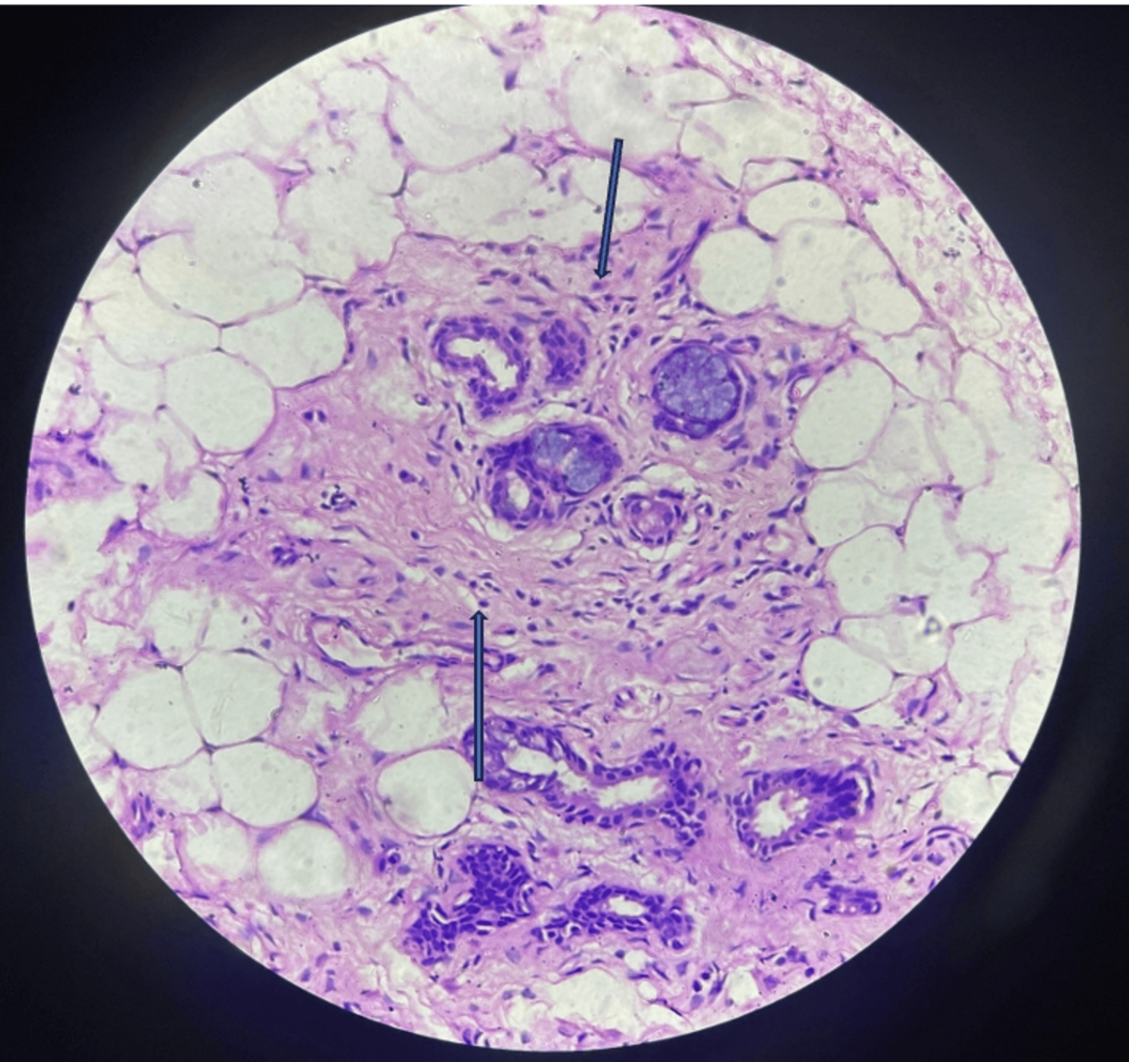
Salivary gland showing inflammatory cells The upper arrow indicates plasma cell, and the lower arrow indicates lymphocyte

Anti-ganglioside panel was negative, ANA by immunofluorescent assay (IFA) was 1+, and Ro-52 antibodies were positive. Lip biopsy was suggestive of chronic sialadenitis suggestive of SS (Figure 2). Schirmer’s test and tear break-up time were normal. The patient was treated with injection methylprednisolone for five days, followed by oral steroids. The patient showed improved gait and eye movements over four weeks.

Case 2

**Figure 3:**
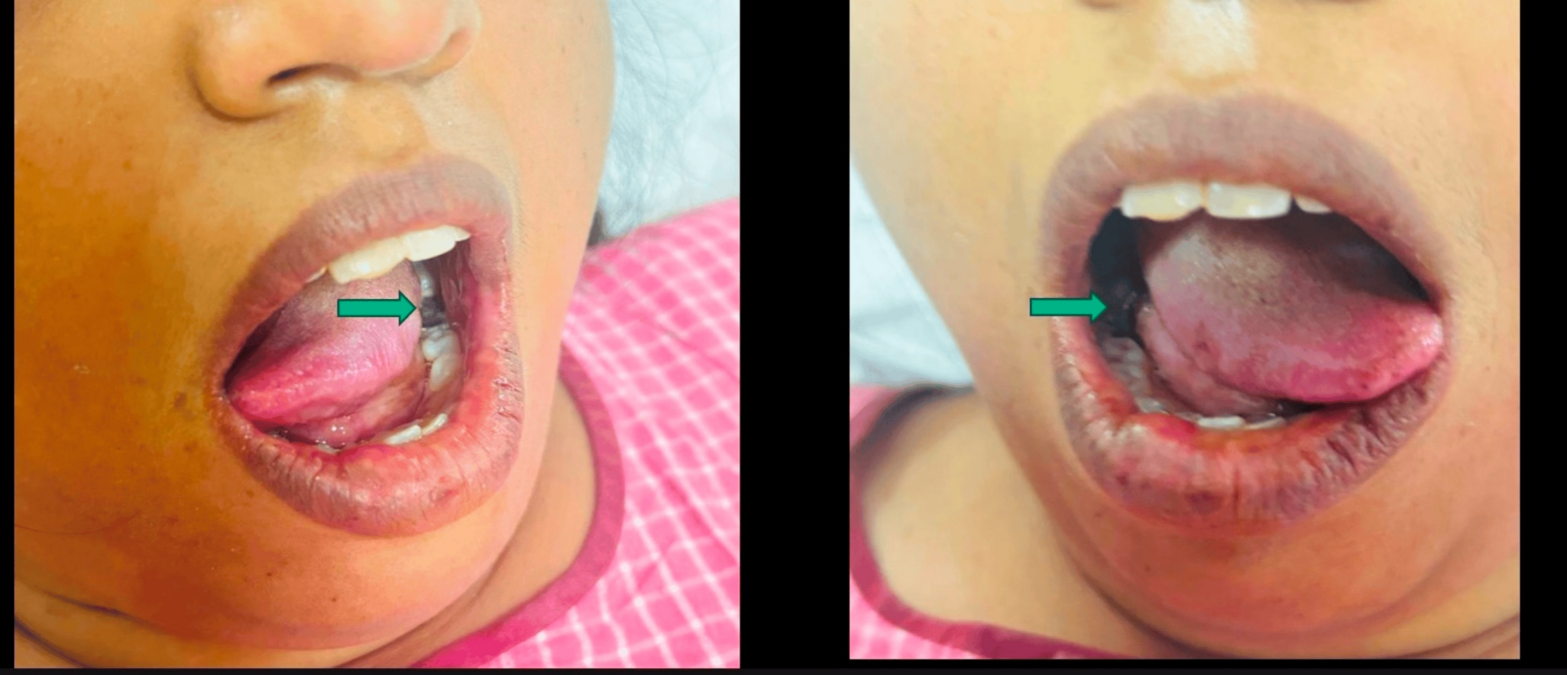
Dental caries due to decreased salivation

A 36-year-old female presented with acute onset tingling and progressive weakness in all four limbs for two days. There was a history of fever three days before the onset of symptoms. On examination, power was grade 4/5 in both lower limbs proximally, with generalized areflexia. Sensory testing and peripheral nerves were normal. Nerve conduction study was suggestive of demyelinating sensorimotor polyneuropathy (Tables 4-6). CSF examination was normal. The patient had a history of five similar episodes in the last six years and was diagnosed with recurrent Guillain-Barre syndrome (GBS), which was treated with intravenous immunoglobulins with complete recovery. We conducted a sural nerve biopsy, which was suggestive of demyelinating neuropathy without onion bulb formation. During her present admission, ANA by IFA was positive (1+), Ro-52 was positive, and SSB was negative. A lip biopsy confirmed the diagnosis of SS. On inquiry, the patient had a history of multiple dental caries but denied symptoms of dry eyes and mouth (Figure 3). Schirmer’s test and tear break-up time were normal. She was treated with steroids with good recovery and advised long-term immunosuppression.

**Table 4:** Motor nerve conduction study showing demyelinating pattern

Nerve	Site	Latency (ms)	Amplitude (mV)	Velocity (m/s)
Right median	Wrist	6.2	8.1	-
	Elbow	11.1	7.6	44.4
Left median	Wrist	5.2	10	-
	Elbow	10.8	8.9	38.9
Right ulnar	Wrist	5.7	5.8	-
	Elbow	11.4	4.5	42.5
Left ulnar	Wrist	4.7	5.8	-
	Elbow	10.4	5.1	42.5
Right peroneal	Ankle	8.7	1.4	-
	Fibula head	17	0.7	34.9
Left peroneal	Ankle	9.9	2.1	-
	Fibula head	17.8	1.4	36.9
Right tibial	Ankle	7.1	4.1	-
	Popliteal	17.9	2.6	32.4
Left tibial	Ankle	7.4	4.2	-
	Popliteal	17.6	2.4	34.4

**Table 5:** F waves showing prolonged latencies suggestive of demyelination ms- meter per second

Nerve	Site	Latency (ms)
Right median	Wrist	39.4
Left median	Wrist	38.1
Right ulnar	Wrist	34.4
Left ulnar	Wrist	37.4
Right peroneal	Ankle	73.8
Left peroneal	Ankle	70.3
Right tibial	Ankle	77.6
Left tibial	Ankle	74.7

**Table 6:** Sensory nerve conduction study

Nerve	Site	Latency (ms)	Amplitude (mV)	Velocity (m/s)
Right median	Wrist	3.8	10.6	37.3
Left median	Wrist	Not recordable		
Right ulnar	Wrist	Not recordable		
Left ulnar	Wrist	6.4	25.2	32
Right sural	Leg	Not recordable		
Left sural	Leg	Not recordable		

Case 3

**Figure 4:**
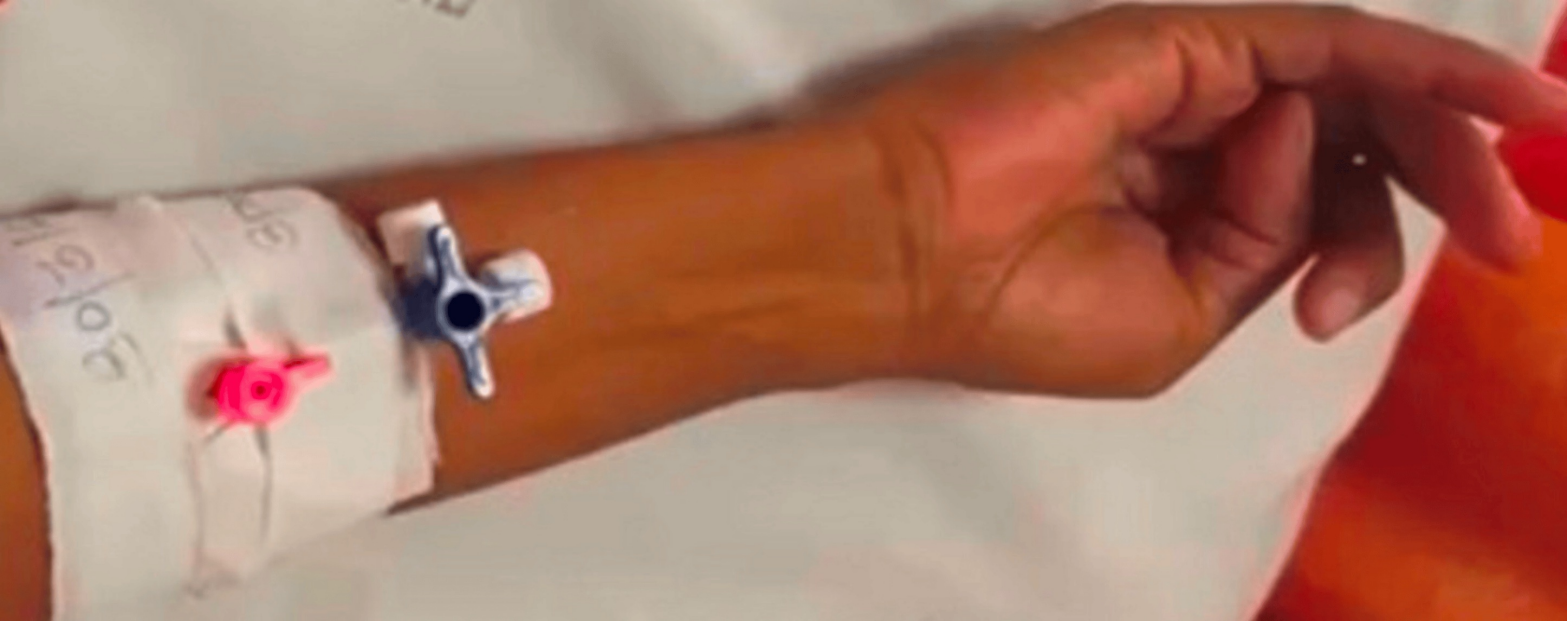
Dystonic posturing of the hand during the episode

A 25-year-old female presented with a history of episodes of abnormal posturing and spasms of all four limbs lasting for 2-3 minutes with 10-20 episodes every day for the last five months (Figure 4).

**Figure 5:**
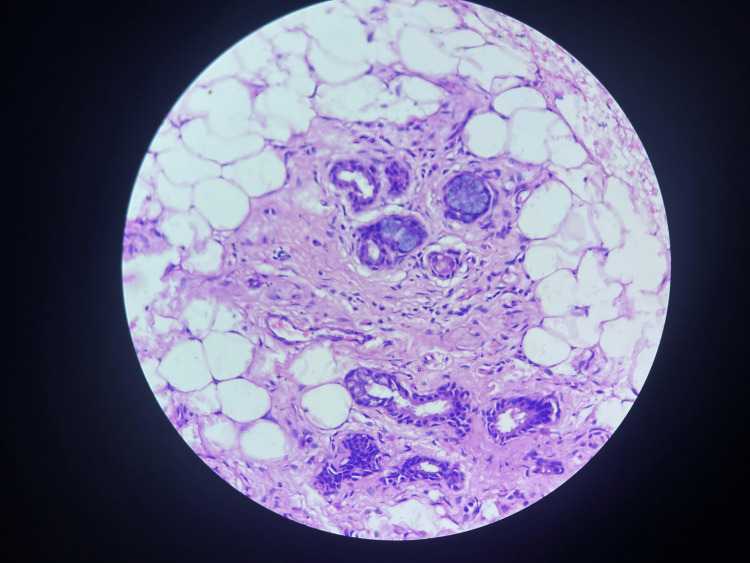
Salivary gland biopsy showing inflammatory infiltrate

On clinical examination, there was no icterus or KF (Kayser-Fleischer) ring, and the liver was not palpable. Neurological examination was normal except for episodes of dystonia. Laboratory investigations, including serum ceruloplasmin, serum iron profile, and ionized calcium levels, were normal. MRI of the brain and spine was also normal. ANA by IFA, anti-Ro-52 (SSA), and anti-La (SSB) were positive. Schirmer’s test and tear break-up time were normal. Lip biopsy was suggestive of SS (Figure 5). The patient was treated with steroids, with good improvement.

Case 4

**Figure 6:**
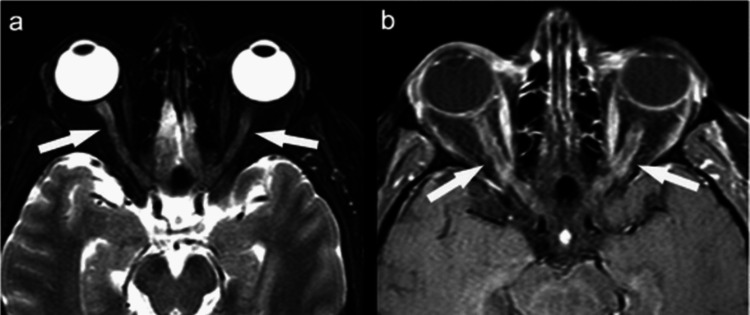
Contrast-enhanced MRI of the orbits showing enhancement of the anterior part of both optic nerves

**Figure 7:**
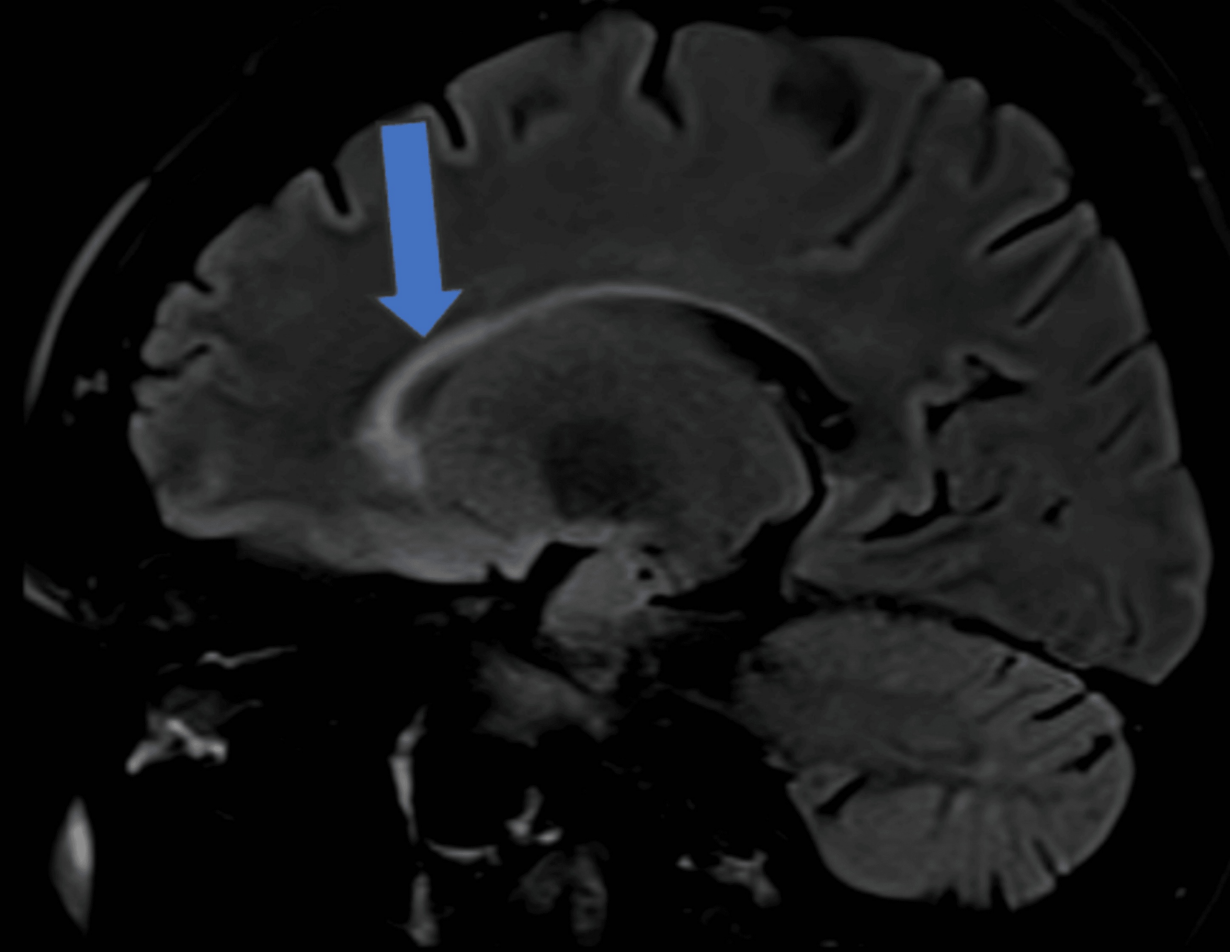
MRI of the brain showing T2 FLAIR hyperintensities in the corpus callosum T2-FLAIR, T2-weighted fluid-attenuated inversion recovery

A 24-year-old female had bilateral optic neuritis in December 2021 (Figure 6). She was found to be positive for myelin oligodendrocyte glycoprotein (MOG) antibodies and was treated with intravenous steroids followed by injection rituximab. She remained asymptomatic for two years when she again presented with symptoms of vertigo and vomiting. MRI of the brain revealed T2 FLAIR hyperintensities in the corpus callosum and subcortical white matter (Figure 7).

**Figure 8:**
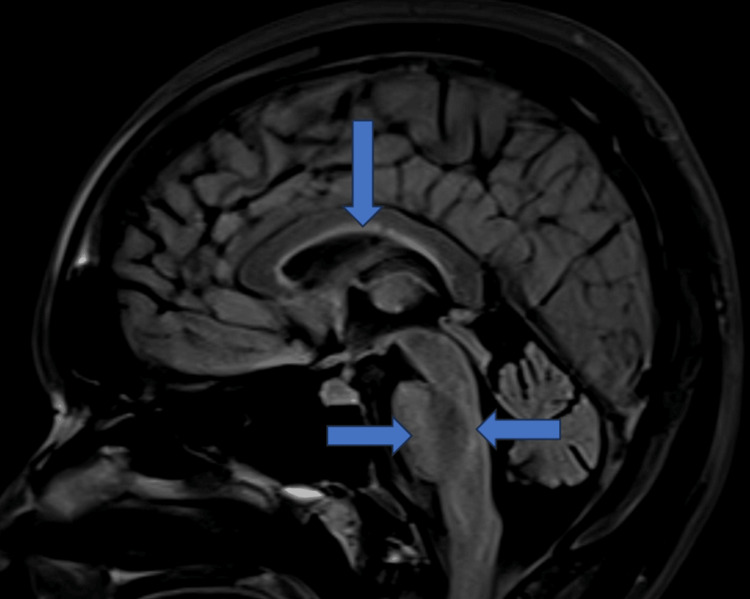
MRI (sagittal) showing T2 FLAIR hyperintensities in the corpus callosum, and periventricular white matter in the pons and medulla (red arrows) T2-FLAIR, T2-weighted fluid-attenuated inversion recovery

Her MOG and aquaporin-4 (AQP-4) antibodies were negative, and she improved with steroids considering demyelination. She remained asymptomatic for six months, after which she presented with a third episode of walking imbalance. MRI of the brain revealed T2 FLAIR hyperintensities in subcortical white matter, corpus callosum, and periventricular regions in the pons and medulla (Figure 8).

**Figure 9:**
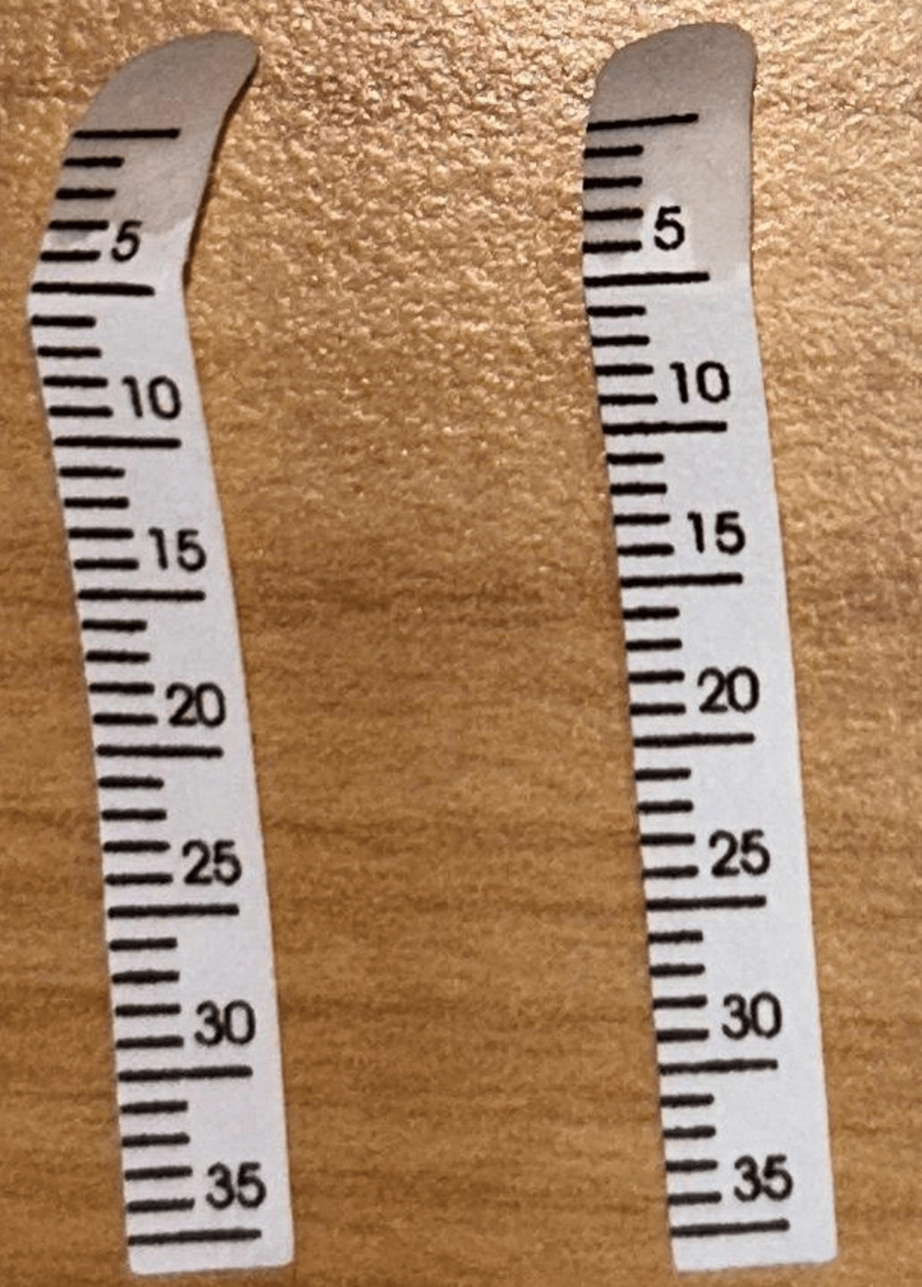
Positive Schirmer tear test

Her MOG and neuromyelitis optica (NMO) antibodies were negative; however, ANA by IFA was positive (2+), and anti-Ro-52 and La antibodies were positive. Schirmer’s test was positive (6 mm), and tear break-up time was normal (Figure 9). She was treated with steroids and plasmapheresis, followed by an injection of rituximab. She had partial improvement in symptoms.

Case 5

**Figure 10:**
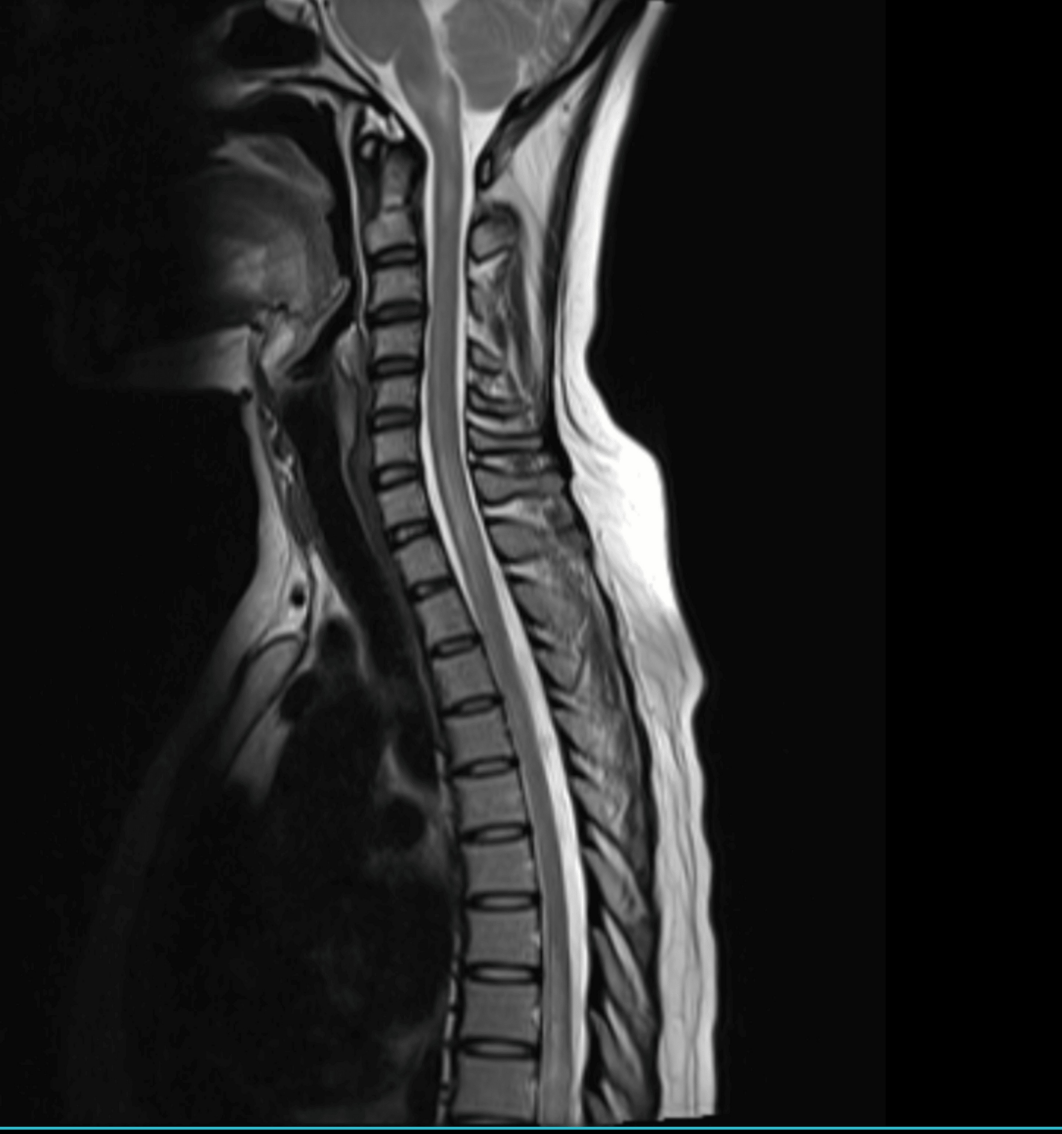
T2-weighted MRI of the spine showing LETM extending from the cervico-medullary junction to the D9 spinal level LETM, longitudinally extensive transverse myelitis

A 28-year-old female presented with left eye optic neuritis with positive NMO antibodies. She was treated with steroids and rituximab. She remained asymptomatic for a year, after which she developed intractable vomiting, hiccups, and sensory motor spastic paraparesis with bladder involvement over 10 days. MRI of the spine revealed LETM extending from the cervico-medullary junction to the D9 level (Figure 10). MRI of the brain revealed T2/FLAIR hyperintensities in periventricular white matter in the fourth ventricle and the right occipital lobe.

CSF examination was normal. ANA by IFA method, anti-SSA (Ro), and anti-SSB (La) antibodies were positive. Schirmer’s test was positive (6 mm). Minor salivary gland biopsy showed focal lymphocytic sialadenitis, which was suggestive of SS. She was treated with plasmapheresis and rituximab. Azathioprine was added as the patient had relapsed on rituximab. The patient made good clinical recovery.

## Discussion

The first case presented with a Miller-Fisher variant of GBS. Only one similar case of Miller-Fisher variant has been described in the literature [5]. Most of the neuropathies in SS are sensory, including small fiber painful neuropathy and ganglionopathy presenting as ataxia.

Our second case presented with recurrent GBS. Primary SS may independently lead to acute demyelinating polyradiculoneuropathy or coexist with GBS. Around 15% of patients with primary SS present with peripheral neuropathy [6]. Although co-occurrence of GBS with SS has been described, recurrent GBS has not been described in the literature [7,8].

Our third case presented with generalized dystonia. Dystonia is a manifestation of the involvement of CNS in SS. Presentation of primary SS with dystonia has been very rarely described [9,10].

The fourth and fifth cases had CNS demyelination. CNS symptoms in SS are either sensorimotor syndromes due to myelitis or visual impairment due to optic neuritis. Active/non-active brain lesions can be seen on MRI. The disease may mimic other primary demyelinating disorders such as multiple sclerosis [11]. The sicca symptoms may be less pronounced in such patients with CNS involvement. The picture gets more confusing when SS occurs in association with other seropositive demyelinating illnesses such as NMO and MOG [12]. Association Of NMO with other systemic autoimmune syndromes including primary SS is well known [13,14]. SS and systemic lupus erythematosus are the most frequently reported systemic autoimmune diseases in patients with neuromyelitis optica spectrum disorder (NMOSD) [15]. Due to the similarity in the protein structure between AQP-4 expressed in the neuronal foot processes and AQP-5 expressed in the salivary glands, inflammation and lymphocytic infiltration are reported in as many as 80% of patients with NMOSD [16]. A systematic review by Guellec et al. highlighted the variable sensitivity (63.9%-85.7%) of the labial minor salivary gland biopsy in diagnosing primary SS despite its high specificity (89.7%-91.9%) for the same [17]. Association of MOGAD with primary SS is very rare, unlike NMO. Only very few such reports are available [18,19].

In our series patients presented with both peripheral and CNS involvement.

## Conclusions

Primary SS is an autoimmune inflammatory disorder that presents with exocrine glandular features and extraglandular features. Extraglandular involvement is uncommon, affecting other systems such as skin, musculoskeletal, respiratory, cardiovascular, and neurological manifestations. Neurological manifestations include involvement of both CNS and peripheral nervous system. The peripheral nervous system may present with axonal sensory or sensorimotor neuropathy, small fiber neuropathy, ganglionopathy, mononeuritis multiplex, multiple cranial neuropathies, or demyelinating radiculoneuropathy such as GBS. CNS involvement may present with optic neuritis, asymptomatic MRI lesions, multiple sclerosis, or transverse myelitis especially LETM. We should have a high degree of suspicion for SS when other usual causes are excluded in typical neurological presentation.
